# Analysing the SPAD dynamics of water-stressed vs. well-watered sesame (*Sesamum indicum* L.) accessions and establishing their relationship with seed yield

**DOI:** 10.7717/peerj.14711

**Published:** 2023-01-18

**Authors:** Lora Anusha Pallavolu, Ratnakumar Pasala, Ramesh Kulasekaran, Brij Bihari Pandey, Umamahesh Virupaksham, Sandhyarani Perika

**Affiliations:** 1 Department of Crop Production, ICAR- Indian Institute of Oilseeds Research, Hyderabad, Telangana, India; 2 Department of Plant Physiology, Acharya N.G. Ranga Agricultural University, Tirupati, Andhra Pradesh, India

**Keywords:** Sesame, SPAD chlorophyll readings, Seed yield, Deficit moisture stress, Correlation and regression

## Abstract

**Background:**

The chlorophyll content is susceptible to deficit moisture stress and may affect the plant yield. Leaf chlorophyll content is directly related to tolerance and higher productivity under deficit moisture stress (WS). The SPAD meter is an excellent tool for rapid analysis of crop chlorophyll content. Therefore, establishing a relationship between leaf chlorophyll content and seed yield is crucial in sesame, particularly under deficit moisture stress.

**Methods:**

Seeds of 37 sesame genotypes with checks were used in this study. These genotypes were mostly landraces, adapted to different agro-ecological zones in India. The selected genotypes were evaluated under well water (WW) and deficit moisture stress (WS) conditions. The SPAD readings were recorded ten (10) times each at every seven days intervals from the juvenile/first bud (30–35 days after sowing) to ripening/ physiological maturity (95–100 days after sowing) stage. This study aimed to identify the association between leaf SPAD readings (recorded at 7-days interval) and seed yield of sesame genotypes.

**Results:**

The analysis of variance revealed the presence of significant variation in SPAD readings due to treatment (WW and WS), genotypes, and their interaction effects. The SPAD readings at all stages were positively correlated with seed yield in both WW and WS. High values of correlation coefficients were observed at 52 (r: 0.672) and 59 (r: 0.655) DAS under WS; whereas at 59 (r: 0.960), 66 (r: 0.972) and 73 (r: 0.974) DAS under WW at one percent significance level (*p* < 0.01), which coincided with the mid-bloom stage of the sesame crop. The best-fit multiple regression model revealed that the dependence of sesame seed yield is significantly influenced by SPAD reading at 52 DAS under WS and 59 to 73 DAS under WW. Both these models provide a good fit with the chi-squared test, which compares the predicted and observed yield.

## Introduction

Sesame is one of the oldest and most important edible oilseed crops in the world due to its high-quality oil, and high content of unsaturated fatty acids (Islam et al., 2017), antioxidants, and protein. Hence, it is widely used for human nutrition, medicinal, and industrial purposes in many countries (Bastug et al., 2021). Sesame is frequently cultivated in arid and semi-arid areas inhabiting “hungry and thirsty” environments (Ramesh et al., 2019), where it is frequently exposed to deficit moisture stress (Ratnakumar & Ramesh, 2019; Pandey et al., 2021a). In rainfed ecosystems, time and duration of water stress have a significant effect on sesame productivity (Ravitej et al., 2019), although the genotypes might have varied responses in maintaining the leaf chlorophyll content and are directly related to tolerance and higher productivity under WS (Kadkhodaie et al., 2014; Pandey et al., 2021b).

Chlorophyll is the most important photosynthetic pigment for capturing light and driving electron transport in reaction centers (Gao et al., 2020). Higher leaf chlorophyll content ensures high photosynthesis and the production of a greater amount of carbohydrates, which in turn increases the plant’s height, number of branches, and seed yield (Nosheen et al., 2019). The SPAD reading is closely correlated with leaf chlorophyll content (Akter et al., 2016; Bunphan, Knoll & Anderson, 2019).

Balanced fertilization is one of the prerequisites for high productivity since sesame demands all essential nutrients (Ramesh, Patra & Biswas, 2017; Ramesh et al., 2019; and Ramesh et al., 2020). Of which nitrogen (N) is a key constituent in chlorophyll structure, requires a sufficient supply of nitrogen for cell division and cell enlargement, larger leaf area, dry matter, and seed yield (Ibrahim et al., 2014; Babajide & Oyeleke, 2014; Zenawi & Mizan, 2019; Ramesh et al., 2021). However, nitrogenous fertilizers improve the leaf chlorophyll content of sesame (Nosheen et al., 2019), deficit moisture stress interferes with the absorption and transfer of nutrients in the crop (Askari et al., 2017).

Establishing the relationship of chlorophyll with seed yield in sesame was very limited. Hence, attempts were made (i) to study the chlorophyll dynamics in sesame and to establish its relationship with seed yield of sesame genotypes under WW and WS through correlation analysis and (ii) to know the dependence of yield on sesame chlorophyll content with multiple regression analysis.

## Materials and Methods

### Plant materials and growth conditions

Seeds of 37 sesame genotypes (IC 132171, IC 132186, IC 132293, IC 204079, IC 204085, IC 204090, IC 204099, IC 204137, IC 204139, IC 204156, IC 204159, IC 204167, IC 204194, IC 204300, IC 204622, IC 205496, IC 205610, IC 205649, IC 205671, IC 205724, IC 205730, IC 205757, IC 205776, IC 205787, IC 205791, IC 205804, JCS 1020, JCS 2454, JCS DT 112, JCS DT 26, JCS DT 97, YLM 66) with two national checks (GT-10 and TKG-22) and a local check (Swetha Til) were used in this study. These genotypes were mostly landraces, adapted to different agro-ecological zones in India. The selected genotypes have similar phenology and maturity duration; whereas checks are better seed yielders under well watered (WW) conditions. The experiment was conducted during the summer season of the year 2021–22 at the ICAR-Indian Institute of Oilseeds Research farm, Narkhoda, Hyderabad, India. Geographically, the research farm is located at 17°15′16″N,˝ 78°18 30E:˝ 542 m above sea level and classified as a semi-arid region in India. The particle size distribution for surface soil had sand: 72.8%, silt: 9.8%, and clay: 17.4% for 0–15 cm, whereas for 15–30 cm, soil had sand: 59.3%, silt: 12.0%, and clay: 25.4%. The experimental soil had a low status of N: 235 kg ha^−1^, P: 9.5 kg ha^−1^, sufficient K: 280 kg ha^−1^, low in SOC: 0.4%, and water holding capacity (18%).

### Crop management

The experiment was conducted in a strip-block design with two treatments, *i.e.,* well watered (WW) and deficit moisture stress (WS). During the experimentation the different water treatments (WW and WS) were considered as factor A and whereas the 35 genotypes were considered as factor B. The seeds were sown in 4 × 3 m^2^ plots at a spacing of 45 cm (row) ×15 cm (between plants) by dibbling seeds in the holes made with khurpi, and replicated thrice. Telangana state’s recommended blanket dose of fertilizers was applied (40:20:20 kg of N: P_2_O_5_: K_2_O/ha^−1^). The P and K were applied as basal doses; while nitrogen was applied in two split doses at basal and 30 DAS. Additional standard crop management practices and need-based plant protection measures were followed to maintain a healthy crop.

### Moisture stress imposition

In WS plots, a deficit moisture stress condition was imposed from the juvenile/first bud (30–35 days after sowing) to ripening/physiological maturity (95–100 days after sowing) stage. Soil moisture levels were recorded by installing six solar-based real-time soil moisture sensors (Proximal; SoilSenS, Model: V1.0, Owings Mills, MD, USA) on an hourly basis at a soil depth of 45 cm. Need-based irrigation was given to WW plots to maintain soil moisture up to 80% of field capacity. In WS plots, stress was imposed up to 45 to 50% soil moisture of the field capacity by withholding the irrigation, equivalent to −4.5 to −5.0 bars of soil water potential.

### Recording of SPAD reading and yield

The SPAD chlorophyll meter (SPAD-502 Plus, make: Konica Minolta, Inc., Toyko, Japan) was used for recording the chlorophyll content among the sesame genotypes. A total of ten plants were selected randomly in each replication under both the WW and WS treatments. The third leaf from the top of the plant (photosynthetically active leaf) was selected for recoding the SPAD readings during the sunny days from 9:30 to 11:30 h IST. The SPAD readings were recorded ten (10) times each at every seven days intervals, *i.e.,* 38 DAS (end of the vegetative stage); 45 and 52 DAS (early bloom stage); 59, 66, 73, and 80 DAS (mid-bloom stage); 87 DAS (late bloom stage); 94 and 101 DAS (physiological maturity stage).

The crop was harvested and seeds were dried for optimum moisture content (6–8%), and then the weights of seeds from five plants in each genotype were recorded in grams. The mean value of the weights was regarded as the seed yield plant^−1^.

### Statistical analysis

Mean values were used for statistical analysis. Analysis of variance (ANOVA), multiple correlation analysis, and multiple regression analysis were performed using IBM SPSS Statistics 28 (IBM Corp., Armonk, NY, USA). The significance of the correlations between different parameters was determined by Pearson’s correlation.

## Results

### Analysis of variance

The analysis of variance (ANOVA) for SPAD chlorophyll meter readings at 10 day intervals, *i.e.,* 38, 45, 52, 59, 66, 73, 80, 87, 94, and 101 DAS; and seed yield of sesame genotypes under both well watered (WW) and deficit moisture stress (WS) conditions were compiled and presented in Table 1. It is evident that seed yield significantly varied due to treatments (WW and WS), genotypes, and their interaction effects, as indicated by one percent level (*p* < 0.01) of significance.

**Table 1:** Analysis of variance (ANOVA) of 35 sesame genotypes for 10 SPAD readings at 7 days interval and yield under well watered and deficit moisture stress conditions.

**Source of variation**	**DF**	**Mean sum of squares**										
		**SPAD 38**	**SPAD 45**	**SPAD 52**	**SPAD 59**	**SPAD 66**	**SPAD 73**	**SPAD 80**	**SPAD 87**	**SPAD 94**	**SPAD 101**	**Yield**
**Factor A (Treatments)**	1	69.21^***^	1.69	666.79^***^	123.74	154.8^*^	2.07	2.30	94	14.14	6.24	37.01^***^
Rep * Factor A **(Error I)**	2	9.77	5.19	2.03	31.42	13.09	27.48	7.16	75.12^***^	15.55	7.26	0.15
**Factor B** **(Genotypes)**	34	12.13^***^	13.95^***^	27.23	19.43	73.25^***^	30.84^***^	30.09	30.1^**^	36.22	48.49^***^	8.3^***^
Rep * Factor B **(Error II)**	68	6.25	6.17	22.4	25.8	26.49^*^	11.16	23.87	18.16	25.7	19.64	0.13
Factor A * Factor B (Interaction)	34	4.72	16.2^**^	21.36	25.05	49.3^***^	28.26^***^	31.4^*^	30.84^***^	20.9	33.97	15.87^***^
**Error III**	68	8.26	9.47	22.65	27.63	18.2	11.26	20.98	15.15	21	29.36	0.13

**Notes.**

*, ** and *** indicate significance at 10, 5 and 1%, respectively.

Variations in SPAD values due to treatments (factor A) were found significant at 38, 52 (*p* < 0.01), and 66 DAS (*p* < 0.1). The variations in the SPAD readings were also observed among 35 genotypes (factor B). In this regard, variations in SPAD values were recorded at 38, 45, 66, 73, 101 (*p* < 0.01), and 87 DAS (*p* < 0.05). In the case of interaction effect (factor A * factor B), variations in SPAD readings at 66, 73, and 87 DAS were significant at one percent level, whereas at 45 and 80 DAS at five and ten percent level, respectively.

### Correlation between SPAD chlorophyll readings and seed yield under WS and WW

The correlation matrix of seed yield and SPAD chlorophyll meter readings of sesame genotypes at different growth stages (7-day interval) under WW and WS is presented in Tables 2 and 3 respectively. The sample size used for the study (n) is 35 genotypes with their corresponding SAPD values at seven days intervals.

**Table 2:** Correlation matrix of seed yield and SPAD readings in sesame genotypes at different growth stages under well watered.

	**Y**	**X**_**1**_	**X**_**2**_	**X**_**3**_	**X**_**4**_	**X**_**5**_	**X**_**6**_	**X**_**7**_	**X**_**8**_	**X**_**9**_	**X**_**10**_
**Y**	1										
**X**_**1**_	.746	1									
**X**_**2**_	.859^*^	0.628	1								
**X**_**3**_	.897^**^	0.667	0.946	1							
**X**_**4**_	.960^***^	0.746	0.767	0.811	1						
**X**_**5**_	.972^***^	0.692	0.815	0.859	0.946	1					
**X**_**6**_	.974^***^	0.702	0.805	0.86	0.952	0.958	1				
**X**_**7**_	.896^***^	0.682	0.912	0.937	0.805	0.854	0.872	1			
**X**_**8**_	.845^*^	0.603	0.653	0.714	0.864	0.81	0.842	0.695	1		
**X**_**9**_	.788^*^	0.812	0.688	0.72	0.814	0.725	0.740	0.655	0.827	1	
**X**_**10**_	.555	0.398	0.626	0.523	0.548	0.443	0.576	0.48	0.558	0.567	1

**Notes.**

Y = Seed yield (g/plants) and X_1_, X_2_, X_3_, X_4_, X_5_, X_6_, X_7_, X_8_, X_9_ and X_10_ are the SPAD readings at 38, 45, 52, 59, 66, 73, 80, 87, 94 and 101 DAS, respectively.

*, ** and *** indicate significance at 10, 5 and 1%, respectively.

**Table 3:** Correlation matrix of seed yield and SPAD values in sesame genotypes at different growth stages under deficit moisture stress.

	**Y**	**X**_**1**_	**X**_**2**_	**X**_**3**_	**X**_**4**_	**X**_**5**_	**X**_**6**_	**X**_**7**_	**X**_**8**_	**X**_**9**_	**X**_**10**_
**Y**	1										
**X**_**1**_	0.309	1									
**X**_**2**_	0.489^*^	0.375	1								
**X**_**3**_	0.672^**^	0.596	0.746	1							
**X**_**4**_	0.655^***^	0.521	0.398	0.548	1						
**X**_**5**_	0.624^***^	0.387	0.603	0.864	0.558	1					
**X**_**6**_	0.606^**^	0.670	0.702	0.952	0.576	0.842	1				
**X**_**7**_	0.604^**^	0.28	0.812	0.814	0.567	0.827	0.740	1			
**X**_**8**_	0.554^**^	0.672	0.692	0.946	0.443	0.810	0.958	0.725	1		
**X**_**9**_	0.464^*^	0.737	0.695	0.919	0.482	0.802	0.954	0.720	0.948	1	
**X**_**10**_	0.387	0.794	0.682	0.805	0.480	0.695	0.872	0.655	0.854	0.928	1

**Notes.**

Y = Seed yield (g/plants) and X_1_, X_2_, X_3_, X_4_, X_5_, X_6_, X_7_, X_8_, X_9_ and X_10_ are the SPAD readings at 38, 45, 52, 59, 66, 73, 80, 87, 94 and101 DAS, respectively.

*, ** and *** indicate significance at 10, 5 and 1%, respectively.

As evident from the values of correlation coefficient (r), the SPAD readings at all stages were positively correlated with seed yield under WW. Correlation coefficient (r) values, which represent the degree of association between SPAD readings and seed yield, were found to continuously increase from 38 to 73 DAS and later decrease from 80 to 101 DAS. High correlation coefficient values with a high level of significance (*p* < 0.01) observed at 59 (r: 0.960), 66 (r: 0.972), and 73 (r: 0.974) DAS of SPAD readings with seed yield. These days in sesame indicate the mid-bloom stage, *i.e.,* 53 to 81 DAS.

Similar to WW, under WS the degree of correlation (r) between SPAD readings and seed yield revealed an increasing trend from 45 to 66 DAS, whereas a decreasing trend from 73 to 101 DAS. In addition, high correlation coefficients with a high level of significance (*p* < 0.01) were detected at 52 (r: 0.672) and 59 (r: 0.655) DAS, which coincide with the end of early bloom and mid-bloom stages of the crop.

### Multiple regression analysis under WW

Under WW conditions, different regression equations were formulated by including SPAD readings from 38 to 101 DAS following the forward selection method. The yield was taken as a dependent variable, while SPAD readings at different periods were taken as independent variables.

Y = −48.65 + 1.61 X_1_ (adjusted *R*^2^ = 0.5434)

Y = −48.46 + 0.71 X_1_ + 0.87 X_2_ (adjusted *R*^2^ = 0.7968)

Y = −38.89 + 0.58 X_1_ + 0.13 X_2_ + 0.44 X_3_ (adjusted *R*^2^ = 0.8303)

Y = −27.28 + 0.04 X_1_ + 0.12 X_2_ + 0.17 X_3_ + 0.38 X_4_ (adjusted *R*^2^ = 0.9597)

Y = −32.01 + 0.10 X_1_ + 0.11 X_2_ + 0.10 X_3_ + 0.20 X_4_ + 0.35 X_5_ (adjusted *R*^2^ = 0.9739)

Y = −26.90 + 0.12 X_1_ + 0.14 X_2_ + 0.04 X_3_ + 0.12 X_4_ + 0.24 X_5_ + 0.10 X_6_ (adjusted *R*^2^ = 0.9798)

Y = −28.64 + 0.11 X_1_ + 0.12 X_2_ + 0.02 X_3_ + 0.13 X_4_ + 0.24 X_5_ + 0.09 X_6_ + 0.08 X_7_ (adjusted *R*^2^ = 0.9794)

Y = −30.29 + 0.12 X_1_ + 0.13 X_2_+ 0.01 X_3_ + 0.10 X_4_ + 0.25 X_5_ + 0.08 X_6_ + 0.07 X_7_ + 0.04 X_8_ (adjusted *R*^2^ = 0.9796)

Y_1_ = −29.80 + 0.10 X_1_ + 0.13 X_2_ + 0.01 X_3_ + 0.09 X_4_ + 0.26 X_5_ + 0.08 X_6_ + 0.09 X_7_ + 0.03 X_8_ + 0.01 X_9_ (adjusted *R*^2^ = 0.9789)

Y_1_ = −32.91 + 0.10 X_1_ + 0.07 X2 + 0.02 X3 + 0.09 X4 + 0.30 X5 + 0.07 X_6_ + 0.12 X_7_ + 0.03 X_8_ + 0.01 X_9_ + 0.05 X_10_ (adjusted *R*^2^ = 0.9783)

Where, Y = Seed yield (g/plant) and X_1_, X_2_, X_3_, X_4_, X_5_, X_6_, X_7_, X_8_, X_9_ and X_10_ are the SPAD readings at 38, 45, 52, 59, 66, 73, 80, 87, 94 and 101 DAS, respectively.

**Figure 1:**
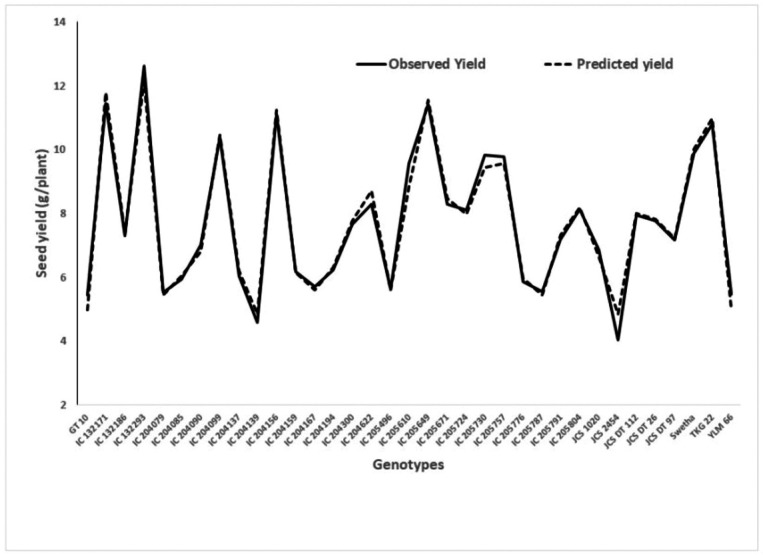
Graphical presentation of observed and predicted seed yield under well watered derived from the regression model.

Based on adjusted R^2^, the equation containing six variables (X_1_ to X_6_) was arbitrated as the best model under WW. From this multiple regression model, a high significance (*p* < 0.01) for SPAD chlorophyll readings at 66 and 73 DAS was observed, which corresponded to the mid-bloom stage of the crop. This model also provides a good fit in the chi-squared test. A graph showing the predicted yield and observed yield under WW is presented in Fig. 1.

### Multiple regression analysis under WS

Similar to the above case, under the WS conditions, various regression equations were formulated by including SPAD readings from 38 to 101 DAS following the forward selection method. The yield was taken as a dependent variable, while SPAD readings at different periods were taken as independent variables.

Y = −3.98 + 0.25 X_1_ (adjusted *R*^2^ = 0.0682)

Y = −11.81 + 0.12 X_1_ + 0.38 X_2_ (adjusted *R*^2^ = 0.2115)

*Y* = 2.97 –0.12 X_1_ – 0.04 X_2_ + 0.18 X_3_ (adjusted *R*^2^ = 0.4144)

Y = −6.46 –0.24 X_1_- 0.05 X_2_ + 0.14 X_3_ + 0.35 X_4_ (adjusted R^2^**=** 0.5698)

Y = −4.81 –0.28 X_1_ – 0.08 X_2_ + 0.19 X_3_ + 0.38 X_4_ – 0.05 X_5_ (adjusted *R*^2^ = 0.5638)

Y = −8.65 – 0.23 X_1_ – 0.07 X_2_ + 0.24 X_3_ + 0.38X_4_ – 0.04 X_5_ – 0.04 X_6_ (adjusted *R*^2^ = 0.5573)

Y = −14.11 – 0.29 X_1_ + 0.09 X_2_ + 0.27 X_3_ + 0.44 X_4_ + 0.01 X_5_ – 0.06 X_6_ – 0.11 X_7_ (adjusted *R*^2^ = 0.5620)

Y = −17.84 – 0.34 X_1_ + 0.11 X_2_ + 0.25 X_3_ + 0.50 X_4_ + 0.01 X_5_ – 0.08 X_6_ – 0.13 X_7_ + 0.12 X_8_ (adjusted *R*^2^ = 0.5513)

Y = −15.51 + 0.08 X_1_ + 0.07 X_2_ + 0.23 X_3_ + 0.22 X_4_ + 0.05 X_5_ + 0.04 X_6_ – 0.02 X_7_ + 0.06 X_8_ – 0.97 X_9_ (adjusted *R*^2^ = 0.6212)

*Y* = 15.54 + 0.09 X_1_ + 0.07 X_2_ + 0.23 X_3_ + 0.23 X_4_ + 0.05 X_5_ + 0.04 X_6_ – 0.01 X_7_ + 0.06 X_8_ – 0.96 X_9_ – 0.01 X_10_ (adjusted *R*^2^ = 0.6055)

Where, Y = Seed yield (g/plants) and X_1_, X_2_, X_3_, X_4_, X_5_, X_6_, X_7_, X_8_, X_9_ and X_10_ are the SPAD readings at 38, 45, 52, 59, 66, 73, 80, 87, 94 and 101 DAS, respectively

Based on adjusted R^2^, the equation containing 9 variables (X_1_ to X_9_) was arbitrated as best model.

**Figure 2:**
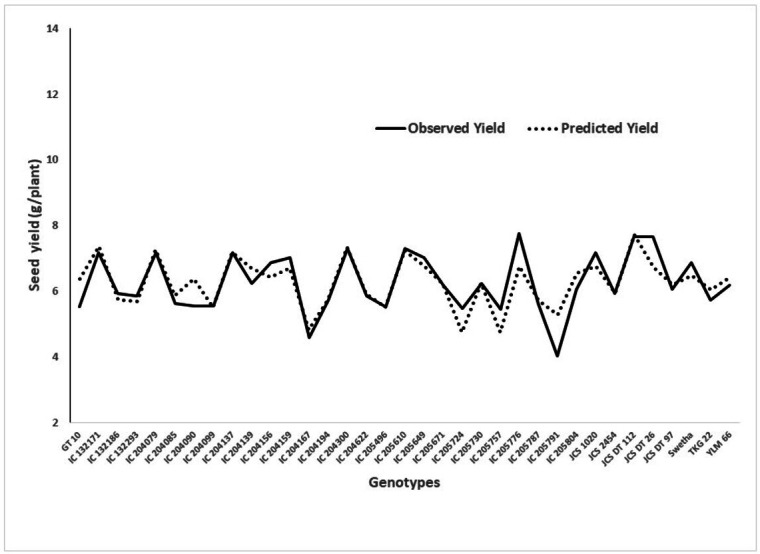
Graphical presentation of observed and predicted seed yield under deficit moisture stress derived from the regression model.

According to this model, the SPAD readings at 52 DAS was found to have high statistical significance (*p* < 0.05) on seed yield. It coincides with the initiation of the mid-bloom stage of the crop, suggesting that maintaining optimum chlorophyll content particularly at the mid-bloom stage could lead to higher yields under WS. This model also provides a good fit in the chi-squared test. A graph showing the predicted yield and observed yield under WW is presented in Fig. 2.

## Discussion

The SPAD reading indicates chlorophyll content (a basic component for photosynthesis) in the leaves (Uddling et al., 2007). The higher leaf chlorophyll content accelerated photosynthetic (Akter et al., 2016) rate and would be a good method for evaluating chlorophyll production (Bunphan, Knoll & Anderson, 2019). The photosynthesis, dry matter production, and yield of sesame is influenced by the chlorophyll content, as it was Nosheen et al. (2019) measured through SPAD value in the study.

Results of the current study suggested that the SPAD readings with seed yield had a high correlation coefficient at 52 and 59 DAS under WS conditions. However, the degree of association was less compared to the WW condition, as indicated by r values. This may be due to the reduction or loss of chlorophyll content in pigment due to photo-oxidation (Anjum et al., 2011) and damage to chloroplasts by active oxygen species under WS conditions (Askari et al., 2017).

While under WW, a positive correlation exists between SPAD chlorophyll readings and seed yield at all crop stages of sesame. At 59, 66, and 73 DAS, high correlation coefficients with a high level of significance (*p* < 0.01) were observed. It coincides with the mid-bloom stage of the sesame crop. The results are consistent with Bunphan, Knoll & Anderson (2019), who also noticed higher SPAD chlorophyll readings in sesame at the mid-bloom stage, *i.e.,* 60 and 75 DAS (56.58 and 58.88) with *p* < 0.01 significance.

Along similar lines, Akter et al. (2016) reported that there was less chlorophyll in the leaves of sesame at the early stages of crop growth. As crop growth progressed, the amount of chlorophyll in leaves increased at a parabolic rate, peaking at about the mid-bloom stage (57 DAS) and subsequently decreasing. Reduced SPAD readings in later stages could be due to leaf senescence and maturity, which had already happened in the reproductive stage. They stated that differences in genetic components and environmental impact might account for the observed variation in SPAD value between genotypes. The ANOVA in the current study is similar to that in the research study of Pandey et al. (2021b) in sesame, which emphasized that the mean performance of SPAD under both WS and WW conditions exhibited significant differences among the genotypes. The selected genotypes in the mentioned studies were from various origins and agro-ecological zones and indicate variations in SPAD values.

The total chlorophyll content is sensitive to deficit moisture stress in various sesame genotypes (Kadkhodaie et al., 2014; Sravanthi et al., 2022). At physiological levels, sesame adopts a few mechanisms that allow the plant to endure WS, including stomatal closure, cuticle thickening, reduced transpiration, prevention of protein depletion, and osmotic management (Hassanzadeh et al., 2009; Ratnakumar et al., 2021).

The multiple regression analysis further confirmed that the seed yield of sesame is highly dependent on the leaf chlorophyll content under both WW and WS. Along similar lines, Nosheen et al. (2019) observed that the seed yield of sesame is influenced by leaf chlorophyll content. The higher rates of photosynthesis are associated with increased carbohydrate production, which in turn promotes the plant’s growth components such as plant height, number of branches, and seed yield. It is understood that the yielding ability of the most important quantitative characteristic in crops depends on the development of other characters (Ramesh et al., 2002).

In the current study, the reduction of chlorophyll content under WS was consistent with reports by Lima et al. (2018) and Bastug et al. (2021), who also confirmed that the WS conditions lead to the destruction of chlorophyll and loss of pigments. Plants with lower chlorophyll levels would be more vulnerable to harm from high light intensities reaching the photosystem and eventually causing damage, resulting in restrictions on growth, development, and production. Under WS conditions, plants use the stomatal closure mechanism to defend themselves from water losses through transpiration, which further contributes to impaired photosynthetic rate and yield (Taiz & Zeiger, 2009).

Another plausible reason for reduced chlorophyll content under WS is that WS conditions promote the production of reactive oxygen species (ROS), which results in the loss of chlorophyll (Bastug et al., 2021). In addition, slower synthesis and faster breakdown or dissociation of the chlorophyll molecules leads to a reduction in chlorophyll content under WS (Kadkhodaie et al., 2014). Under WS, it could be opined that considering chlorophyll content as a trait may help to increase light interception and conversion efficiency and thus maintain or increase crop yields in sesame (Anjum et al., 2011; Kapoor et al., 2020).

The results of the present study were also supported by Sehgal et al. (2021), who also suggested that management of sesame crops for optimal yields under varying growing conditions requires an understanding of the key levels of leaf N and the functional relationships between leaf N, growth and developmental processes. In their study, leaf photosynthesis declined at 31 DAS more than stomatal conductance and transpiration rates, indicating that both stomatal and non-stomatal mechanisms are involved in the decrease in photosynthesis in response to leaf N. In addition, Mehmood et al. (2021) suggested that the nitrogen uptake or contents of sesame leaves were highest at the mid-bloom stage (65 DAS) and lowest at the physiological maturity stage. This could be due to the higher biomass accumulation and partitioning at the mid-bloom stage (beginning of seed filling stage), which contributes to increasing seed yield in sesame (Couch et al., 2017).

In the present study, maiden attempt have been made to understand the chlorophyll dynamics in sesame and to establish its relationship with seed yield for the chosen sesame genotypes under WS and WW. In addition, efforts were made to probe the question of whether and to what extent chlorophyll status in leaves imparts drought tolerance in sesame.

## Conclusion

Chlorophyll is one of the most important chloroplast components for photosynthesis because it harvests light and produces reducing power. Photosynthetic activities gets reduced under deficit moisture stress due to the damage of chlorophyll components, which leads to reduced crop yields. Though crop yield is affected by multiple factors, chlorophyll content plays a pivotal role in determining the yield under deficit moisture stress. Results of the present study, as indicated by the correlation analysis, revealed that SPAD readings were highly correlated and showed a high level of significance (*p* < 0.01) with seed yield at 52 and 59 days after sowing under deficit moisture stress, whereas under well watered, similar natured correlation coefficients were observed at 59, 66, and 73 days after sowing. Multiple regression analysis revealed that, the dependence of yield on SPAD chlorophyll readings was statistically significant (*p* < 0.05) at 52 days after sowing under deficit moisture stress. Alternatively, under well watered, the dependence of yield was found to be statistically significant (*p* < 0.01) at 59, 66, and 73 days after sowing. These periods coincide with the mid-bloom stage of the sesame crop. Hence, it can be opined that maintaining higher chlorophyll content during mid-bloom stage through nitrogen supply would be helpful in increasing the crop yields in sesame. It can also be stated that the genotypes which are maintaining high SPAD chlorophyll readings under deficit moisture stress are considered tolerant genotypes under deficit moisture stress and used for the selection process.

##  Supplemental Information

10.7717/peerj.14711/supp-1 Supplemental Information 1 The matric potential (-bars) curve presented in the graph was estimated using the soil samples before the treatments by soil tensiometer The percent of soil moisture (Y axis) derived from real-time soil moisture sensors were fitted against the soil matric potential (X axis) under IR (straight line, where soil moisture was −0.55 bars) and DS (dotted line, where soil moisture was −4.65 bars) conditions. Therefore, it was estimated that the soil matric potential was recorded around −0.55 bars under irrigated, −4.65 bars under stress conditions.

10.7717/peerj.14711/supp-2 Supplemental Information 2 Supplementary Tables

## Additional Information and Declarations

### Data Availability

The following information was supplied regarding data availability: The raw data are available in the Tables.
