# Supplementary material for: Analysing the SPAD dynamics of water-stressed vs. well-watered sesame (Sesamum indicum L.) accessions and establishing their relationship with seed yield
Source: PeerJ. 2023 Jan 18;11:e14711. doi: 10.7717/peerj.14711 (PMC9864184; doi:10.7717/peerj.14711)
Supplement: Supplemental Information 1 — The percent of soil moisture (Y axis) derived from real-time soil moisture sensors were fitted against the soil matric potential (X axis) under IR (straight line, where soil moisture was −0.55 bars) and DS (dotted line, where soil moisture was −4.65 bars) conditions. Therefore, it was estimated that the soil matric potential was recorded around −0.55 bars under irrigated, −4.65 bars under stress conditions. [file peerj-11-14711-s001.docx]

**Supplementary Figure 1.** The matric potential (-bars) curve presented in the graph was estimated using the soil samples before the treatments by soil tensiometer. The percent of soil moisture (Y axis) derived from real-time soil moisture sensors were fitted against the soil matric potential (X axis) under IR (straight line, where soil moisture was -0.55 bars) and DS (dotted line, where soil moisture was -4.65 bars) conditions. Therefore, it was estimated that the soil matric potential was recorded around -0.55 bars under irrigated, -4.65 bars under stress conditions.
