# Supplementary material for: Analysing the SPAD dynamics of water-stressed vs. well-watered sesame (Sesamum indicum L.) accessions and establishing their relationship with seed yield
Source: PeerJ. 2023 Jan 18;11:e14711. doi: 10.7717/peerj.14711 (PMC9864184; doi:10.7717/peerj.14711)
Supplement: Supplemental Information 2 [file peerj-11-14711-s002.docx]

**Supplementary Table 1:** Raw data of SPAD readings at 10 different intervals under Water Stress (WS)

| **Sl no.** | **Genotypes** | **38 DAS** | **45 DAS** | **52 DAS** | **59 DAS** | **66 DAS** | **73 DAS** | **80 DAS** | **87 DAS** | **94 DAS** | **101 DAS** | **YIELD** |
| --- | --- | --- | --- | --- | --- | --- | --- | --- | --- | --- | --- | --- |
| 1 | IC 205776 | 40.12 | 35.32 | 54.56 | 46.10 | 55.16 | 50.56 | 34.30 | 47.40 | 46.60 | 50.52 | 7.76 |
| 2 | JCS DT 26 | 40.12 | 35.32 | 54.52 | 46.10 | 54.94 | 50.42 | 34.30 | 47.36 | 46.60 | 50.52 | 7.65 |
| 3 | JCS DT 112 | 42.00 | 37.66 | 62.00 | 49.42 | 62.10 | 62.12 | 45.28 | 51.23 | 49.42 | 54.52 | 7.65 |
| 4 | IC 204300 | 41.16 | 34.68 | 54.26 | 49.42 | 54.84 | 48.76 | 34.30 | 46.20 | 46.60 | 50.18 | 7.31 |
| 5 | IC 205610 | 41.25 | 37.48 | 61.00 | 49.02 | 61.32 | 61.00 | 43.42 | 51.00 | 49.42 | 54.46 | 7.31 |
| 6 | IC 204079 | 41.16 | 34.68 | 54.02 | 49.42 | 54.84 | 48.62 | 34.30 | 46.00 | 46.60 | 50.18 | 7.18 |
| 7 | IC 132171 | 41.22 | 37.38 | 60.00 | 49.02 | 61.20 | 60.75 | 42.94 | 50.73 | 49.02 | 54.32 | 7.18 |
| 8 | IC 204137 | 40.92 | 34.68 | 53.94 | 49.02 | 54.54 | 48.44 | 34.30 | 45.89 | 46.50 | 50.18 | 7.18 |
| 9 | JCS 1020 | 44.00 | 37.10 | 59.00 | 48.42 | 50.00 | 60.25 | 35.56 | 50.45 | 49.02 | 54.24 | 7.18 |
| 10 | IC 204159 | 40.92 | 34.30 | 52.14 | 49.02 | 54.52 | 48.44 | 34.30 | 45.30 | 46.50 | 49.70 | 7.02 |
| 11 | IC 205649 | 43.75 | 35.32 | 58.00 | 48.42 | 59.00 | 60.00 | 35.12 | 50.30 | 49.02 | 54.52 | 7.02 |
| 12 | IC 204156 | 40.60 | 34.30 | 51.88 | 48.42 | 53.88 | 48.30 | 34.30 | 45.00 | 46.50 | 49.70 | 6.87 |
| 13 | Swetha | 43.25 | 34.68 | 57.00 | 49.02 | 58.00 | 59.00 | 35.00 | 49.70 | 49.02 | 54.46 | 6.87 |
| 14 | IC 205730 | 40.60 | 34.30 | 51.44 | 48.42 | 53.88 | 47.32 | 34.30 | 44.23 | 46.50 | 49.70 | 6.23 |
| 15 | IC 204139 | 43.20 | 34.68 | 56.00 | 49.02 | 57.00 | 58.00 | 34.92 | 49.10 | 48.42 | 54.32 | 6.23 |
| 16 | IC 205671 | 40.12 | 35.32 | 50.98 | 47.90 | 53.56 | 46.80 | 34.30 | 43.84 | 46.26 | 50.70 | 6.17 |
| 17 | YLM 66 | 43.00 | 34.68 | 55.92 | 48.42 | 56.50 | 57.00 | 34.72 | 48.64 | 48.42 | 54.24 | 6.17 |
| 18 | JCS DT 97 | 40.12 | 35.32 | 50.98 | 47.90 | 53.14 | 46.80 | 34.00 | 43.84 | 46.26 | 50.70 | 6.06 |
| 19 | IC 205804 | 42.94 | 37.38 | 55.86 | 48.42 | 56.30 | 56.00 | 34.68 | 48.48 | 48.42 | 53.24 | 6.06 |
| 20 | JCS 2454 | 40.12 | 35.32 | 50.88 | 47.14 | 52.86 | 46.00 | 34.00 | 43.64 | 46.25 | 50.52 | 5.93 |
| 21 | IC 132186 | 41.16 | 35.21 | 55.72 | 46.60 | 56.20 | 55.00 | 34.68 | 48.18 | 48.42 | 53.14 | 5.93 |
| 22 | IC 204622 | 40.12 | 34.68 | 50.88 | 47.14 | 52.52 | 46.00 | 33.00 | 43.64 | 46.25 | 50.52 | 5.86 |
| 23 | IC 132293 | 41.16 | 35.32 | 55.72 | 46.60 | 56.00 | 54.00 | 34.30 | 47.96 | 48.42 | 53.10 | 5.86 |
| 24 | IC 204194 | 41.16 | 34.68 | 50.72 | 46.60 | 51.92 | 40.78 | 33.00 | 43.84 | 46.12 | 50.52 | 5.72 |
| 25 | TKG 22 | 40.92 | 34.68 | 55.70 | 46.50 | 55.96 | 53.00 | 34.30 | 47.84 | 47.90 | 50.70 | 5.72 |
| 26 | IC 205787 | 41.16 | 34.68 | 50.72 | 46.60 | 51.80 | 40.78 | 33.00 | 43.84 | 46.12 | 50.52 | 5.63 |
| 27 | IC 204085 | 40.92 | 34.68 | 55.28 | 46.50 | 55.34 | 52.00 | 34.30 | 47.80 | 47.90 | 50.70 | 5.63 |
| 28 | IC 204099 | 40.92 | 34.30 | 50.24 | 46.50 | 50.82 | 40.78 | 33.00 | 43.64 | 46.10 | 50.18 | 5.55 |
| 29 | IC 204090 | 40.60 | 34.68 | 54.84 | 46.26 | 55.32 | 51.00 | 34.30 | 47.76 | 47.14 | 50.52 | 5.55 |
| 30 | IC 205496 | 40.92 | 34.30 | 50.24 | 46.50 | 50.82 | 40.78 | 32.00 | 43.64 | 46.10 | 50.18 | 5.53 |
| 31 | GT 10 | 40.60 | 35.32 | 54.70 | 46.26 | 55.28 | 50.80 | 34.30 | 47.72 | 47.14 | 50.52 | 5.53 |
| 32 | IC 205724 | 40.60 | 34.30 | 46.92 | 46.26 | 50.76 | 40.26 | 32.00 | 43.84 | 46.00 | 50.18 | 5.48 |
| 33 | IC 205757 | 40.60 | 34.16 | 46.92 | 46.26 | 50.76 | 40.26 | 31.45 | 43.84 | 46.00 | 49.70 | 5.46 |
| 34 | IC 204167 | 40.12 | 34.16 | 46.92 | 46.10 | 48.42 | 40.26 | 31.12 | 43.64 | 45.70 | 49.70 | 4.59 |
| 35 | IC 205791 | 40.12 | 34.16 | 46.92 | 46.10 | 48.42 | 40.26 | 31.12 | 43.64 | 45.25 | 49.70 | 4.03 |

**Supplementary Table 2:** Raw data of SPAD readings at 10 different intervals under Well Water (WW)

| Sl no. | **Genotypes** | **38 DAS** | **45 DAS** | **52 DAS** | **59 DAS** | **66 DAS** | **73 DAS** | **80 DAS** | **87 DAS** | **94 DAS** | **101 DAS** | **YIELD** |
| --- | --- | --- | --- | --- | --- | --- | --- | --- | --- | --- | --- | --- |
| 1 | IC 205776 | 37.66 | 45.40 | 54.88 | 62.00 | 51.23 | 62.12 | 54.52 | 62.10 | 45.28 | 49.42 | 12.63 |
| 2 | JCS DT 26 | 37.48 | 45.33 | 53.28 | 61.00 | 51.00 | 61.00 | 54.46 | 61.32 | 43.42 | 49.02 | 11.43 |
| 3 | JCS DT 112 | 37.38 | 45.28 | 53.18 | 60.00 | 50.73 | 60.75 | 54.32 | 61.20 | 42.94 | 49.02 | 11.41 |
| 4 | IC 204300 | 37.10 | 45.23 | 52.62 | 59.00 | 50.45 | 60.25 | 54.24 | 50.00 | 35.56 | 48.42 | 11.10 |
| 5 | IC 205610 | 35.32 | 45.40 | 54.88 | 58.00 | 50.30 | 60.00 | 54.52 | 59.00 | 35.12 | 48.42 | 10.81 |
| 6 | IC 204079 | 34.68 | 45.33 | 53.28 | 57.00 | 49.70 | 59.00 | 54.46 | 58.00 | 35.00 | 49.02 | 10.37 |
| 7 | IC 132171 | 34.68 | 45.28 | 51.62 | 56.00 | 49.10 | 58.00 | 54.32 | 57.00 | 34.92 | 49.02 | 9.87 |
| 8 | IC 204137 | 34.68 | 43.42 | 49.56 | 55.92 | 48.64 | 57.00 | 54.24 | 56.50 | 34.72 | 48.42 | 9.82 |
| 9 | JCS 1020 | 37.38 | 42.94 | 49.28 | 55.86 | 48.48 | 56.00 | 53.24 | 56.30 | 34.68 | 48.42 | 9.78 |
| 10 | IC 204159 | 35.21 | 41.16 | 49.22 | 55.72 | 48.18 | 55.00 | 53.14 | 56.20 | 34.68 | 46.60 | 9.57 |
| 11 | IC 205649 | 35.32 | 41.16 | 49.16 | 55.72 | 47.96 | 54.00 | 53.10 | 56.00 | 34.30 | 46.60 | 8.29 |
| 12 | IC 204156 | 34.68 | 40.92 | 49.06 | 55.70 | 47.84 | 53.00 | 50.70 | 55.96 | 34.30 | 46.50 | 8.29 |
| 13 | Swetha | 34.68 | 40.92 | 46.44 | 55.28 | 47.80 | 52.00 | 50.70 | 55.34 | 34.30 | 46.50 | 8.14 |
| 14 | IC 205730 | 34.68 | 40.60 | 46.44 | 54.84 | 47.76 | 51.00 | 50.52 | 55.32 | 34.30 | 46.26 | 8.10 |
| 15 | IC 204139 | 35.32 | 40.60 | 46.02 | 54.70 | 47.72 | 50.80 | 50.52 | 55.28 | 34.30 | 46.26 | 7.95 |
| 16 | IC 205671 | 35.32 | 40.12 | 46.02 | 54.56 | 47.40 | 50.56 | 50.52 | 55.16 | 34.30 | 46.10 | 7.76 |
| 17 | YLM 66 | 35.32 | 40.12 | 45.50 | 54.52 | 47.36 | 50.42 | 50.52 | 54.94 | 34.30 | 46.10 | 7.65 |
| 18 | JCS DT 97 | 34.68 | 41.16 | 45.50 | 54.26 | 46.20 | 48.76 | 50.18 | 54.84 | 34.30 | 49.42 | 7.31 |
| 19 | IC 205804 | 34.68 | 41.16 | 46.44 | 54.02 | 46.00 | 48.62 | 50.18 | 54.84 | 34.30 | 49.42 | 7.18 |
| 20 | JCS 2454 | 34.68 | 40.92 | 46.44 | 53.94 | 45.89 | 48.44 | 50.18 | 54.54 | 34.30 | 49.02 | 7.18 |
| 21 | IC 132186 | 34.30 | 40.92 | 46.02 | 52.14 | 45.30 | 48.44 | 49.70 | 54.52 | 34.30 | 49.02 | 7.02 |
| 22 | IC 204622 | 34.30 | 40.60 | 46.02 | 51.88 | 45.00 | 48.30 | 49.70 | 53.88 | 34.30 | 48.42 | 6.87 |
| 23 | IC 132293 | 34.30 | 40.60 | 45.50 | 51.44 | 44.23 | 47.32 | 49.70 | 53.88 | 34.30 | 48.42 | 6.23 |
| 24 | IC 204194 | 35.32 | 40.12 | 45.50 | 50.98 | 43.84 | 46.80 | 50.70 | 53.56 | 34.30 | 47.90 | 6.17 |
| 25 | TKG 22 | 35.32 | 40.12 | 46.44 | 50.98 | 43.84 | 46.80 | 50.70 | 53.14 | 34.00 | 47.90 | 6.06 |
| 26 | IC 205787 | 35.32 | 40.12 | 46.02 | 50.88 | 43.64 | 46.00 | 50.52 | 52.86 | 34.00 | 47.14 | 5.93 |
| 27 | IC 204085 | 34.68 | 40.12 | 46.02 | 50.88 | 43.64 | 46.00 | 50.52 | 52.52 | 33.00 | 47.14 | 5.86 |
| 28 | IC 204099 | 34.68 | 41.16 | 45.50 | 50.72 | 43.84 | 40.78 | 50.52 | 51.92 | 33.00 | 46.60 | 5.72 |
| 29 | IC 204090 | 34.68 | 41.16 | 45.50 | 50.72 | 43.84 | 40.78 | 50.52 | 51.80 | 33.00 | 46.60 | 5.63 |
| 30 | IC 205496 | 34.30 | 40.92 | 46.44 | 50.24 | 43.64 | 40.78 | 50.18 | 50.82 | 33.00 | 46.50 | 5.55 |
| 31 | GT 10 | 34.30 | 40.92 | 46.44 | 50.24 | 43.64 | 40.78 | 50.18 | 50.82 | 32.00 | 46.50 | 5.53 |
| 32 | IC 205724 | 34.30 | 40.60 | 46.02 | 46.92 | 43.84 | 40.26 | 50.18 | 50.76 | 32.00 | 46.26 | 5.48 |
| 33 | IC 205757 | 34.16 | 40.60 | 46.02 | 46.92 | 43.84 | 40.26 | 49.70 | 50.76 | 31.45 | 46.26 | 5.46 |
| 34 | IC 204167 | 34.16 | 40.12 | 45.50 | 46.92 | 43.64 | 40.26 | 49.70 | 48.42 | 31.12 | 46.10 | 4.59 |
| 35 | IC 205791 | 34.16 | 40.12 | 45.50 | 46.92 | 43.64 | 40.26 | 49.70 | 48.42 | 31.12 | 46.10 | 4.03 |
